# Association Between *NR3C1* Mutations and Glucocorticoid Resistance in Children With Acute Lymphoblastic Leukemia

**DOI:** 10.3389/fphar.2021.634956

**Published:** 2021-03-29

**Authors:** Haiyan Liu, Ziping Li, Fei Qiu, Chunjie Li, Xiaojing Lin, Yingyi He, Maoxiang Qian, Yuanbin Song, Hui Zhang

**Affiliations:** ^1^ Department of Hematology/Oncology, Guangzhou Women and Children’s Medical Center, Guangzhou, China; ^2^ Institute of Pediatrics, Guangzhou Women and Children’s Medical Center, Guangzhou, China; ^3^ Bioinspired Engineering and Biomechanics Center, Xi’an Jiaotong University, Xi’an, China; ^4^ Institute of Pediatrics and Department of Hematology and Oncology, Children's Hospital of Fudan University, National Children's Medical Center, the Shanghai Key Laboratory of Medical Epigenetics, International Co-laboratory of Medical Epigenetics and Metabolism, Ministry of Science and Technology, Institutes of Biomedical Sciences, Fudan University, Shanghai, China; ^5^ State Key Laboratory of Oncology in South China, Department of Hematologic Oncology, Sun Yat-sen University Cancer Center, Collaborative Innovation Center for Cancer Medicine, Guangzhou, China

**Keywords:** glucocorticoid, glucocorticoid receptor, drug resistance, NR3C1, acute lymphoblastic leukemia

## Abstract

Treatment outcomes in children with acute lymphoblastic leukemia (ALL) have been improved substantially, with a cure rate exceeding 80% using conventional therapy. However, the outcome for patients with relapsed/refractory ALL remains unsatisfactory, despite the fact that these patients generally receive more intense therapy. Glucocorticoid (GC) resistance is a leading cause of treatment failure and relapse in ALL. Abnormal *NR3C1* transcription and/or translation is strongly associated with GC resistance, but the underlying molecular mechanism and the clinical value of NR3C1 alterations with GC resistance in ALL treatment remain unclear. This study applied panel sequencing to 333 newly diagnosed and 18 relapsed ALL samples to characterize the link between NR3C1 and ALL further. We identified *NR3C1* mutations in three patients with newly diagnosed ALL (0.9%) and two patients with relapsed ALL (11.1%). Functional analyses revealed that four of these five *NR3C1* mutations (p. R477H, p. Y478C, p. P530fs, and p. H726P) were loss-of-function (LoF) mutations. A drug sensitivity test further showed that LoF *NR3C1* mutations influence GC resistance. Saturated mutagenesis of hotspot R477 demonstrated the importance of this residue for NR3C1 function. The dominant-negative effect of p. R477C and p. R477S and the non-dominant negative effect of p. R477H and p. Y478C suggests multiple mechanisms underlying GC resistance. Thus, primary or acquired genomic lesions in *NR3C1* may play a critical role in GC resistance and contribute to ALL treatment failure and/or relapse.

## Introduction

Acute lymphoblastic leukemia (ALL), a highly aggressive hematologic malignancy, is the most common cancer in children. After a 60 years' endeavor, ALL is now considered a highly curable cancer in children with contemporary therapeutic regimens (Pui et al., 2018). However, relapse remains a challenge, since it is the leading cause of treatment failure and subsequent death, and relapsed ALL has poor prognosis. There is substantial evidence indicating that ALL relapse is mainly due to multi-drug resistance (Choi et al., 2007; Iacobucci and Mullighan, 2017). For example, Bhojwani et al. demonstrated that the treatment of relapsed ALL is becoming increasingly challenging due to resistance to chemotherapy (Bhojwani and Pui, 2013; Pierro et al., 2017). Additionally, mutations induced by chemotherapy may cause drug resistance and lead to ALL relapse (Brown and Ferrando, 2018).

As the first agents introduced for the treatment of ALL, glucocorticoids (GCs) are the most effective and frequently used agents for childhood ALL. They are essential drugs not only for ALL but also for other lymphoid malignancies (Hunger and Mullighan, 2015). Various findings support the importance of GCs in ALL. First, the clinical response to prednisone is a strong predictor of overall treatment outcomes in children with ALL. Prednisone good responder is associated with a favorable prognosis in ALL, while prednisone poor responder is usually strongly associated with an increased risk of recurrence (Gao and Liu, 2018). Second, dexamethasone (DEX), a type of GC, can pass the blood-brain barrier and penetrate the central nervous system to cure and/or prevent central nervous system leukemia (Zhou et al., 2019). Third, GC resistance contributes significantly to induction failure or relapse in ALL (Paugh et al., 2015). Finally, drug sensitivity assays have shown that GC resistance *in vitro* is associated with unfavorable outcomes (Meyer et al., 2020).

The advent of next-generation sequencing (NGS) has contributed to the identification of genetic factors related to GC resistance in ALL, including mutations involved in IKAROS family zinc finger 1 (*IKZF1*) (Marke et al., 2016), BTG Anti-Proliferation Factor 1 (*BTG1*) (Scheijen et al., 2017), TBL1X Receptor 1 (*TBL1XR1*) (Jones et al., 2014), CREB binding protein (*CREBBP*) (Mullighan et al., 2011), and nuclear receptor subfamily 3 group C member 1 (*NR3C1*). Among these, *NR3C1* mutations are the most frequent genetic events contributing to GC resistance (Li et al., 2020). *NR3C1*, encoding glucocorticoid receptor (GR), functions as a transcription activator by binding to glucocorticoid response elements that are mainly found in the promoters of glucocorticoid-responsive genes. Thus, the disruption of GR expression and function influences the therapeutic effect of GCs in patients with ALL. Abnormalities in NR3C1, such as decreased expression and loss of protein function, contribute to the differential expression of alternatively spliced and translated GR isoforms and are the key to GC resistance (Swierczewska et al., 2013). Li et al. identified seven functional *NR3C1* mutations (p.S114fs, p. E285X, p.M336fs, p. LC456–457HS, p. R477C, p. R477H, and p. R714Q) related to ALL relapse and GC resistance (Li et al., 2020). However, the mechanisms by which NR3C1 mutations affect resistance have not been determined. Thus, a systemic study of *NR3C1* mutations in ALL relapse may drive the generation of novel therapies.

In this study, we screened and functionally examined *NR3C1* mutations in primary and relapsed ALL samples from patients enrolled in the CCCG-2015-ALL study in our hospital. The results may drive the improvement of clinical outcomes and help produce a generation of novel therapies for ALL.

## Methods

### Patients

Patients with newly diagnosed (*N* = 333) and relapsed (*N* = 18) B-ALL were enrolled. This study was approved by the Ethics Committees of Guangzhou Women and Children’s Medical Center (2015020936, 2017102307) and was conducted in accordance with the ethical guidelines of the Declaration of Helsinki. According to institutional guidelines, informed consent was obtained from the parents or guardians.

### Cell Lines

293T cells, Nalm6 cells (*DUX4*-*IGH* rearrangement), and REH cells (*ETV6*-*RUNX1* rearrangements) were purchased from the American Type Culture Collection (ATCC, Manassas, VA, United States). 293T and Nalm6 cells were maintained in Dulbecco’s Modified Eagle Medium and RPMI1640 (Invitrogen, Renfrew, United Kingdom) supplemented with 10% fetal bovine serum (Gibco, Waltham, MA, United States). The LookOut® Mycoplasma PCR Detection Kit was used to (Sigma Aldrich, MO, Unitesd States) to confirm the mycoplasma-free status of tested cell line in this study.

### Next-Generation Sequencing and Validation

Panel sequencing of hematological malignancy-related genes (Supplementary Table S1) was performed at Kindstar Global (Beijing) Technology, Inc. DNA samples extracted from salvia samples were used as germline samples. Targeted gene capture and library construction for NGS were performed using NimbleGen Sequence Capture Arrays (Roche, Basel, Switzerland). NGS libraries were sequenced to generate 150-bp paired-end reads on the Illumina HiSeq X10 instrument (San Diego, CA, United States) according to the manufacturer’s protocol. Sequencing reads were aligned to the human reference genome (hg19) using Burrows–Wheeler Aligner (BWA-0.7.10). Duplicated reads were then marked and removed using Picard (picard-tools-2.17.0). Variant calls were performed using VarDictJava (1.5.8) (Lai et al., 2016) with pre-curated blacklist variant filters and custom Annovar scripts. Final confident variants were then annotated using PCGR (Pierro et al., 2017), and tier1 and tier2 variants were manually checked using IGV. Structural variants were called using Delly (Rausch et al., 2012; Hunger and Mullighan, 2015) and filtered using BreakTrans. *NR3C1* mutations were validated by Sanger sequencing with specific primers (Supplementary Table S2).

### *NR3C1* Transcriptional Activity

The full-length *NR3C1* cDNA was cloned into the pCDNA 3.1 expression vector. *NR3C1* mutants were generated using the Q5 Site-Directed Mutagenesis Kit (New England Biolabs, Ipswich, MA, United States) with the primers listed in Supplementary Table S2. The transactivation activity of wild-type NR3C1 and twenty-four mutants (p.R477X, p. R477G, p. R477W, p. R477E, p. R477I, p. R477Y, p. R477K, p. R477S, p. R477A, p. R477D,p.R477F, p. R477H, p. R477P, p. R477Q, p. R477T, p. R477V, p. R477L, p. R477C, p. R477N, p. R477W, p. Y478C, p. P530fs, p. I539fs, and p. H726P) was examined in the presence of 100 nM dexamethasone (DEX) using Cignal Reporter Assay Kits (CCS-006L; Qiagen, Hilden, Germany) following the manufacturer’s protocol. A luciferase reporter assay was performed using the Dual-Luciferase Reporter (DLR) Assay Systems (Promega, Madison, WI, United States). All experiments were performed in triplicate and repeated independently three times.

### Lenti-viral Transfection and Cytotoxicity Assay

Full-length NR3C1 cDNA was amplified and cloned into the cL20c-IRES-GFP lentiviral vector, and mutations were generated using Q5 Site-Directed Mutagenesis Kit (New England Biolabs, United States) with primers listed in Supplementary Table S2. Lentiviral supernatants were produced by transient transfection of HEK-293T cells using Lipofectamine 3,000 (Invitrogen, United Kingdom) following the manufacturer's protocol. Wild-type or mutant *NR3C1* was ectopically overexpressed in Nalm6 cells (*DUX4*-*IGH* rearrangement) and REH cells (*ETV6-RUNX1*) by lentiviral transduction. Briefly, lentiviral particles were spun with 12 ng/ml polybrene for 2 hat 1,200 g, 4°C. Fourty-eight hours later, GFP^+^ cells were sorted using FACSAria™ Cell Sorter (BD) and the transduction was confirmed by western blot (Supplementary Figure S1). All stable cells were seeded at a density of 3 × 10^3^ cells/well in 96-well plates and treated with increasing doses of prednisolone (0, 0.0256, 0.128, 0.64, 3.2, 16, 80, and 400 mM) or DEX (0, 10^–5^, 10^–4^, 10^–3^, 10^–2^, 10^–1^, 1, and 10 μg/ml) for 72 h. Cell viability was measured by the 3-(4,5-dimethylthiazol-2-yl)-2,5-diphenyltetrazolium bromide (MTT) assay using a Multiscan MS spectrophotometer (LabSystems, Stockholm, Sweden). Experiments were performed in triplicate and repeated at least three times.

### Quantitative Real-Time PCR Assay

Total RNA was extracted using the RNeasy Micro Kit (Qiagen) according to the manufacturer’s protocol, and 1 μg of RNA was reverse transcribed into cDNA using the Invitrogen Second Strand cDNA Synthesis Kit with random hexamers (Thermo Fisher Scientific, Waltham, MA, United States). qRT-PCR was performed using the ABI Prism 7900HT Detection System (Applied Biosystems, Foster City, CA, United States) with FastStart SYBR Green Master Mix (Roche). *GAPDH* was used as an internal control. Primers are listed in Supplementary Table S2.

### Statistical Analysis

Data are presented as mean ± SD. All statistical analyses were performed using GraphPad Prism (GraphPad Software, La Jolla, CA) and R (version 3.2.5, https://www.R-project.org). All tests were two-sided Student’s *t*-tests. A *p* value less then 0.05 was considered statistically significant.

## Results

### NR3C1 Alterations in Patients With ALL

**FIGURE 1:**
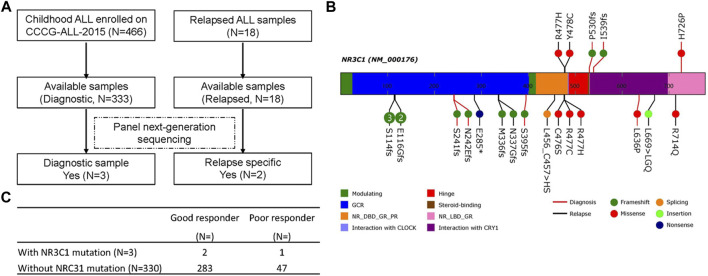
*NR3C1* mutations in patients with newly diagnosed and relapsed B-ALL. **(A)** Identification of NR3C1 mutations by panel sequencing in patients with newly diagnosed (*N* = 333) and relapsed (*N* = 18) B-ALL enrolled in CCCG-ALL-2015 in Guangzhou Women and Children’s Medical Center. **(B)** *NR3C1* mutations data from PCGP, cBioPortal, and our hospital were analyzed. *NR3C1* mutations spanned the full length of the gene (upper: p. R477H, p. Y478C, p. P530fs, p. I539fs, p. H726P from our hospital; lower: p. S114fs, p. E116Gfs, p. S241fs, p. N242Efs, p. E285*, p.M336fs, p. N337Gfs, p. S395fs, p. L456_C457 > HS, p. C476S, p. R477C, p. R477H, p. L639P, p. L669 > LGQ, p. R714Q form PCGP and cBioPortal; red line, newly diagnosed B-ALL; black line, relapsed B-ALL). **(C)** Dexamethasone treatment responses were analyzed in patients with newly diagnosed ALL with (*N* = 3) or without (*N* = 330) an *NR3C1* mutation.

A total of 466 children with ALL were enrolled from March 2015 to June 2020 in the CCCG-ALL-2015 study of Guangzhou Women and Children’s Medical Center, including 18 patients with relapsed ALL. Among these 466 patients, 333 diagnostic DNA samples were available. The baseline characteristics of the ALL cohort are summarized in Tables 1, 2; Supplementary Tables S3, S4. As shown in Figures 1A,B, three patients (0.9%) with *NR3C1* mutations (p.I539fs, p. P530fs, and p. H726P) were identified among 333 patients with newly diagnosed ALL. In the relapsed cohort, 2 of 18 patients (11.1%) had *NR3C1* mutations (p.R477H and p. Y478C) (Figure 1B, upper panel), indicating that *NR3C1* mutations play a more prominent role in relapsed ALL. The early GC response is an important index reflecting treatment outcomes. Interestingly, one out of three patients with newly diagnosed ALL carrying an *NR3C1* mutation responded poorly to DEX treatment, which means that there is no obvious effective cytoreduction after 8 days of DEX therapy. Meanwhile, 47 newly diagnosed patients without *NR3C1* mutations responded poorly to DEX treatment (Figure 1C). To systemically evaluate *NR3C1* mutations in children with ALL, we retrieved *NR3C1* mutation data from pediatric cancer genome project (Downing et al., 2012) and cBioPortal (Puente et al., 2011), as summarized in Figure 1B (lower panel). We found that *NR3C1* was frequently mutated in the glucocorticoid receptor (GCR) domain, DNA-binding domain (DBD), and ligand-binding domain (LBD).

**TABLE 1:** Baseline characteristics of newly diagnosed ALL patients with or without NR3C1 mutations.

Characteristic		Without *NR3C1* mutations	With *NR3C1* mutations	*p* Value
	Case number (*N* = )	330	3	
Age (%)	> = 1, = <10	301 (91.2)	3 (100.0)	1
	<1, >10	29 (8.8)	0 (0.0)	
Gender (%)	Female	128 (38.8)	0 (0.0)	0.288
	Male	202 (61.2)	3 (100.0)	
WBC (%)	<50 × 10^9^/L	261 (79.6)	1 (33.3)	0.111
	> = 50 × 10^9^/L	67 (20.4)	2 (66.7)	
Liver (%)	<2 cm	159 (48.2)	1 (33.3)	0.698
	> = 2, <5 cm	143 (43.3)	2 (66.7)	
	> = 5 cm	28 (8.5)	0 (0.0)	
Spleen (%)	<2 cm	205 (62.1)	2 (66.7)	1
	> = 2, <5 cm	104 (31.5)	1 (33.3)	
	> = 5 cm	21 (6.4)	0 (0.0)	
Mediastinal mass (%)	No	324 (98.2)	2 (66.7)	0.062
	Yes	6 (1.8)	1 (33.3)	
FAB subtype (%)	L1	58 (17.7)	0 (0.0)	0.58
	L2	213 (64.9)	1 (50.0)	
	L3	57 (17.4)	1 (50.0)	
Immunophenotype (%)	B-ALL	301 (91.2)	2 (66.7)	0.247
	T-ALL	29 (8.8)	1 (33.3)	
Fusion genes (%)	Not identified	217 (65.8)	0 (0.0)	0.001
	ETV6-RUNX1	67 (20.3)	0 (0.0)	
	E2A-fusion	15 (4.5)	0 (0.0)	
	MLL rearrangement	5 (1.5)	1 (33.3)	
	BCR-ABL	13 (3.9)	0 (0.0)	
	Other fusion	13 (3.9)	2 (66.7)	
CNSL (%)	No	321 (97.6)	3 (100.0)	1
	Yes	8 (2.4)	0 (0.0)	
GC response (%)	Good	283 (85.8)	2 (66.7)	0.374
	Poor	47 (14.2)	1 (33.3)	
D19 MRD (%)	<1%	256 (77.8)	3 (100.0)	1
	> = 1%	73 (22.2)	0 (0.0)	
D46 (%)	> = 0.1%	303 (92.7)	1 (50.0)	0.146
	<0.1%	24 (7.3)	1 (50.0)	
Risk stratification (%)	Low risk	168 (50.9)	0 (0.0)	0.168
	Intermediate risk	155 (47.0)	3 (100.0)	
	High risk	7 (2.1)	0 (0.0)	

**TABLE 2:** Baseline characteristics of relapsed ALL patients with or without NR3C1 mutations.

Characteristic		Without *NR3C1* mutations	With *NR3C1* mutations	*p* Value
	Case number (*N* = )	16	2	
Age (%)	> = 1, = <10	14 (87.5)	0 (0.0)	0.039
	<1, >10	2 (12.5)	2 (100.0)	
Gender (%)	Female	7 (43.8)	1 (50.0)	1
	Male	9 (56.2)	1 (50.0)	
WBC (%)	<50 × 10^9^/L	16 (100.0)	2 (100.0)	NA
Liver (%)	<2 cm	10 (62.5)	1 (50.0)	1
	> = 2, <5 cm	4 (25.0)	1 (50.0)	
	> = 5 cm	2 (12.5)	0 (0.0)	
Spleen (%)	<2 cm	10 (71.4)	1 (100.0)	1
	> = 2, <5 cm	4 (28.6)	0 (0.0)	
	> = 5 cm	16 (100.0)	2 (100.0)	NA
Immunophenotype (%)	B-ALL	16 (100.0)	2 (100.0)	NA
Fusion genes (%)	Not identified	10 (62.5)	1 (50.0)	0.641
	ETV6-RUNX1	3 (18.8)	1 (50.0)	
	E2A-fusion	1 (6.2)	0 (0.0)	
	BCR-ABL	1 (6.2)	0 (0.0)	
	Others	1 (6.2)	0 (0.0)	
BM relapse (%)	No	4 (25.0)	0 (0.0)	1
	Yes	12 (75.0)	2 (100.0)	
CNS relapse (%)	No	13 (81.2)	2 (100.0)	1
	Yes	3 (18.8)	0 (0.0)	
Testis relapse (%)	No	11 (68.8)	2 (100.0)	1
	Yes	5 (31.2)	0 (0.0)	
Combination relapse (%)	No	13 (81.2)	2 (100.0)	1
	Yes	3 (18.8)	0 (0.0)	
Risk stratification (%)	Standard risk	4 (25.0)	0 (0.0)	0.255
	Intermediate risk	11 (68.8)	1 (50.0)	
	High risk	1 (6.2)	1 (50.0)	

NA, not applicable.

### NR3C1 Mutations Confer GC Resistance to ALL Cells

**FIGURE 2:**
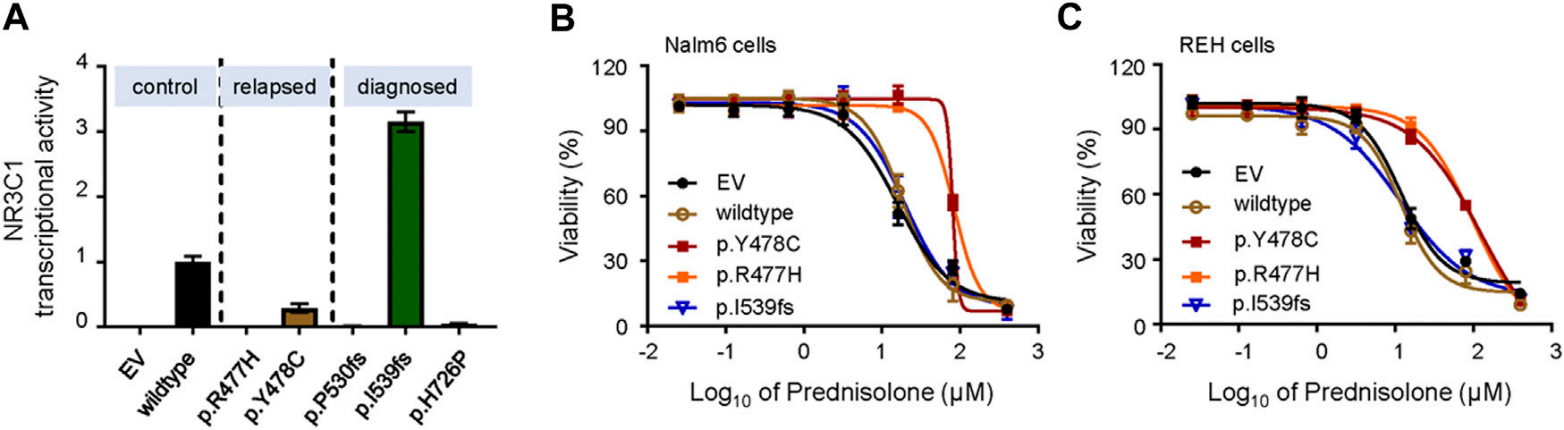
Transcriptional activity and effect on GC resistance for various *NR3C1* mutations in ALL cells. **(A)** The activity of NR3C1 mutants and wild-type NR3C1 treated with dexamethasone was tested by glucocorticoid response element (GRE)-reporter assays (black bar, wild-type; orange bar, p. Y478C; green bar, p. I539fs). **(B)** Cytotoxicity of prednisolone was examined in Nalm6 cells harboring mutant NR3C1 (blue line, EV; brown line, wild-type NR3C1; purple line, p. Y478C; orange line, p. R477H). **(C)** Cytotoxicity of prednisone was examined in REH cells with mutant NR3C1 (blue line, EV; brown line, wild-type NR3C1; purple line, p. Y478C; orange line, p. R477H.). NR3C1 mutations (p. Y478C and p. R477H) lead to significant resistance to prednisolone in ALL lines. Cells were incubated with drugs for 72 h, and viability was then measured by an MTT assay. Experiments were performed in triplicate and repeated at least three times.

To further demonstrate the role of *NR3C1* mutations in GCs response, we cloned wild-type *NR3C1* and its mutant forms, and evaluated the effects of mutations on DEX-induced *NR3C1* transcriptional activity. First, we applied the glucocorticoid response element (GRE)-reporter assay to examine the effects of *NR3C1* mutations on DEX-induced GRE activity. As shown in Figure 2A, transcriptional activity was completely abolished for all relapse-related NR3C1 mutations (p.R477H and p. Y478C). Similar patterns were observed for two mutations detected at initial diagnosis (p.P530fs and p. H726 P), while the p. I539fs mutation did not impair its transcriptional function. Second, we established Nalm6 cells ectopically overexpressing *NR3C1* mutants by lentiviral transfection and tested the effect of mutations on the drug response to GCs. In accordance with the results for transcriptional activity, prednisolone resistance was identified in Nalm6 cells with *NR3C1* mutants (Figure 2B). The 50% inhibitory concentrations (IC_50_) for the NR3C1-Mutant (Y478C, R477H, and I539fs) were 80.3, 84.1, and 19.8 µM, respectively, while the IC_50_ value of prednisone for wild-type Nalm6 cells was only 15.8 µM (Figure 2B). The same response pattern was found in REH cells with *NR3C1* mutations. The IC_50_ values of prednisone were 128.80, 96.89, and 11.7 µM in REH cells with Y478C, R477H, and I539fs, respecitvely, (Figure 2C). Furthermore, the impact of *NR3C1* mutations on GC resistance was confirmed by DEX treatment in both ALL cell lines (Supplementary Figure S2). These findings provide direct evidence for the importance of *NR3C1* in the GC response in ALL cells.

### Saturation Mutagenesis of NR3C1 R477 and its Impact on Wild-type NR3C1

**FIGURE 3:**
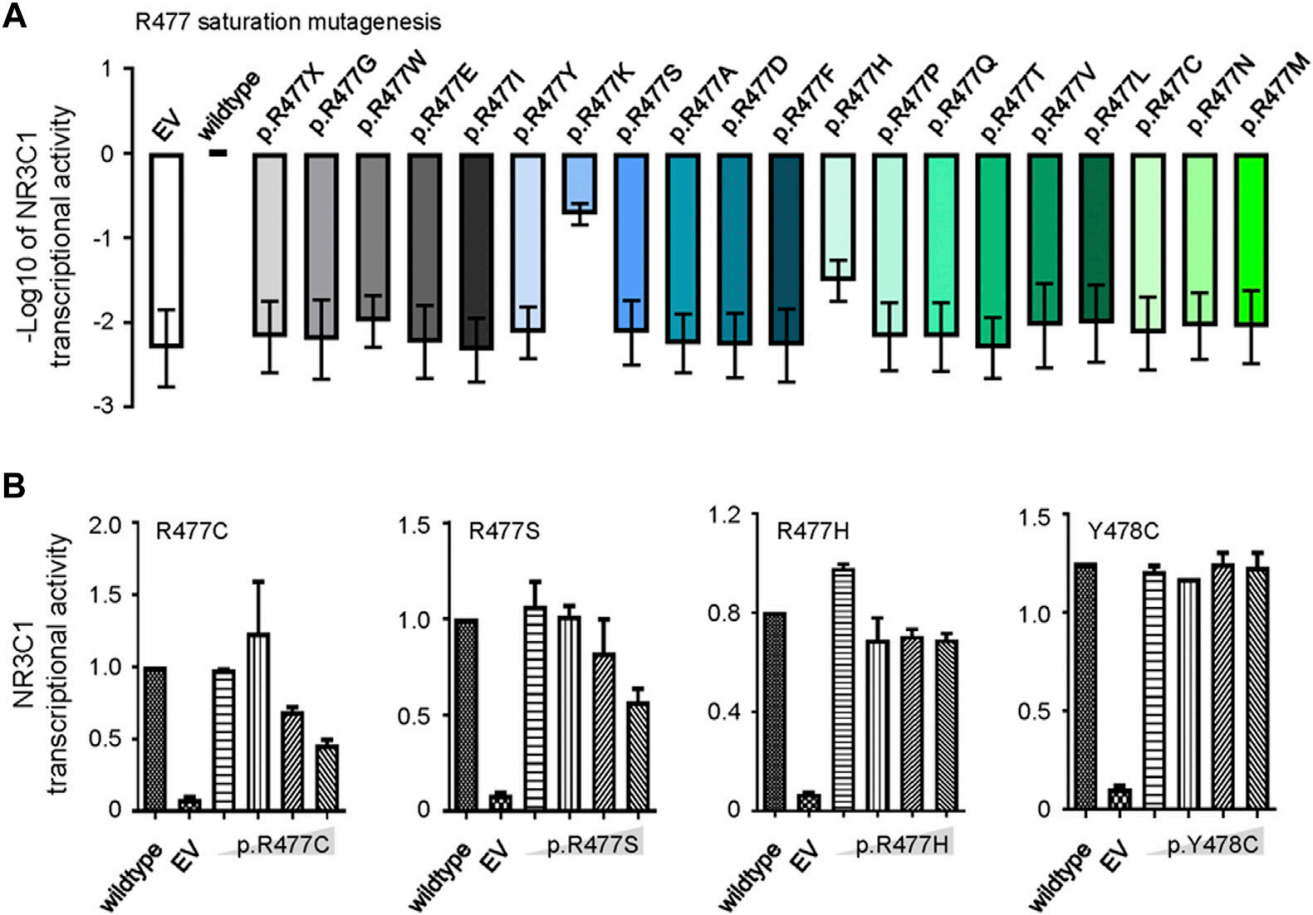
Saturation mutagenesis of NR3C1 R477 and its effects on protein function. **(A)** Twenty R477 mutation types were constructed by saturated mutagenesis. The transcriptional activity of NR3C1 mutations was presented as the −log10 value of relative activity. **(B)** Wild-type and R477 mutant (p.R477C, p. R477S, p. R477H, and p. Y478C) NR3C1 were co-transfected in different proportions (1:1, 1:2, 1:4, and 1:8). Mutated NR3C1 (p.R477C and p. R477S) showed dominant-negative regulatory effects on the transcriptional activity of wild-type NR3C1, while mutant NR3C1 (p.R477H and p. Y478C) showed non-dominant-negative regulatory effects on the transcriptional activity of wild-type NR3C1. All data are presented as means ± SD of triplicate samples from each independent experiment.

Since R477 residue was frequently mutated in this study during ALL relapse (Figure 1B), we determined the precise effects of mutations on the function of NR3C1 and evaluated whether this residue played a dominant functional role. In particular, we used saturated mutagenesis to create all twenty R477 mutants and evaluated their transcriptional activity. As shown in Figure 3A, p. R477C and p. R477 S showed almost complete loss of function (LoF), which is consistent with the results of Li et al. (Marke et al., 2016). More importantly, we observed an LoF pattern for all R477 mutations, with varying impacts from 0.65 to 19.5%, suggesting an essential role of the R477 residue in NR3C1 functions. Next, we evaluated the mutation effects on wild-type NR3C1 transcriptional activity by co-transfection of wild-type NR3C1 with the R477-mutant (p.R477C, p. R477S, and p. R477H) and p. Y478C NR3C1. As shown in Figure 3B, with an increasing dose of NR3C1 mutations (p.R477C and p. R477S), the transcriptional activity of NR3C1 gradually decreased. However, the combination of wild-type NR3C1 with p. R477H and p. Y478C did not negatively affect transcriptional activity, suggesting a distinct effect of the R477 mutation on the wild-type NR3C1 protein.

**FIGURE 4:**
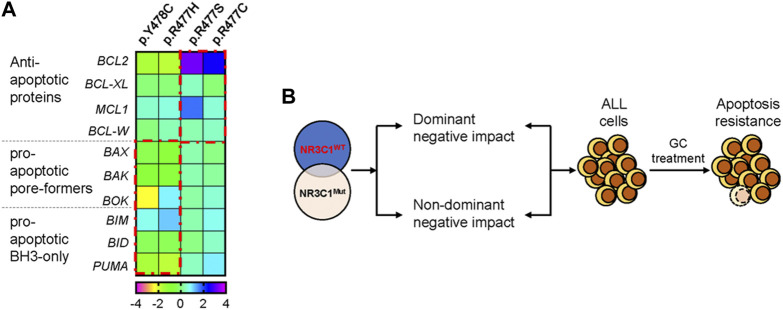
NR3C1 mutations contribute to GC resistance by dominant-negative and non-dominant-negative regulatory effects. **(A)** The expression of apoptosis-related genes in NR3C1 mutants was determined by quantitative PCR. The expression levels of anti-apoptotic genes (*BCL2, BCL-XL, MCL1,* and *BCL-W*) were up-regulated in NR3C1 mutants (p.R477C and R477S) by a dominant-negative regulatory effect. Pro-apoptotic genes (*BAX, BAK, BOK, BIM, BID,* and *PUMA*) were down-regulated in NR3C1 mutants (p.R477H andp. Y478C) by non-dominant-negative regulation. **(B)** Model of the mechanism underlying GC resistance caused by NR3C1 mutations.

Mounting evidence suggests that NR3C1 directly regulates mitochondrial-related apoptotic gene expression by binding to the promoter region. To explore the potential mechanism, we designed real-time quantitative PCR primers for apoptosis-related genes (*BCL2*, *BCL*-*XL*, *MCL1*, *BCL*-*W*, *BAX*, *BAK*, *BOK*, *BIM*, *BID*, and *PUMA*) and evaluated gene expression levels before and after prednisolone treatment. As shown in Figure 4A, the anti-apoptotic genes *BCL2* and *MCL1* were significantly up-regulated in Nalm6 cells with dominant-negative mutations (p.R477C and p. R477S, 5.5 to 9.2-fold up-regulation in *BCL2*, and 1.4 to 3.2-fold up-regulation in *MCL1*) but not in cells with non-dominant-negative mutations (p.R477H and p. Y478C). However, the pro-apoptotic genes *BAX*, *BAK*, *BOK*, *BIM*, *BID*, and *PUMA* were down-regulated in the dominant-negative group, suggesting increased anti-apoptosis and decreased pro-apoptosis mediate *NR3C1* mutation-induced GC resistance (Figure 4B).

## Discussion

ALL is a highly aggressive hematologic malignancy resulting from the transformation of early lymphoid progenitors. GC resistance represents a significant challenge in the treatment of ALL. Generalized GC resistance is characterized by impaired cortisol signaling resulting from mutations in the *NR3C1* gene that encodes GR. However, the mechanisms underlying GC resistance in ALL remain incompletely understood. Recent studies have revealed that relapse-specific mutations in the *NR3C1* gene are frequently detected in ALL patients (El-Fayoumi et al., 2018; Xiao et al., 2019; van der Zwet et al., 2021). In this study, we sequenced 333 newly diagnosed and 18 relapsed ALL samples to further characterize the link between *NR3C1* and ALL. Our data show that *NR3C1* mutations are more frequent in patients with relapsed ALL samples. These results are consistent with those of previous reports, despite the fact that we present a relatively small case number from our study cohort (Xiao et al., 2019; Li et al., 2020).

*NR3C1* mutations are among the most frequent genetic events contributing to GC resistance (Oshima et al., 2016). NR3C1 is composed of an N-terminal transactivation domain, a central DBD, and a C-terminal LBD. Previously reported mutations in *NR3C1* are mainly located in the DBD and LBD (Foussier et al., 2019). Our study found that *NR3C1* mutations spanned the entire gene, although most functional *NR3C1* mutations were located in the DBD and LBD (Figure 1), consistent with previous results (Paragliola et al., 2020). Missense mutations had the highest frequency and were mainly located in the DBD. Therefore, we selected representative mutations in the DBD for further research. Since NR3C1 regulates the target genes’ transcription by forming homodimers or heterodimers and binding to the target gene promoter activation element GRE (Ramamoorthy and Cidlowski, 2013), the homodimer or heterodimer characteristics and the effects of NR3C1 mutants on its function are worth exploring. A previous study has shown that heterozygous missense mutations in *NR3C1* in the N-terminal domain, without dominant-negative effects, exert regular transactivation activity (Foussier et al., 2019). Our MTT assay showed that NR3C1 transcription activity was negatively correlated with GC resistance (Figure 2). Thus, we tested the dominant-negative regulatory effect by a luciferase gene reporter experiment and found two protein-protein regulation modes in the DBD domain, namely dominant-negative regulation (p.R477C and p. R477S) and non-dominant-negative regulation (p.R477H and p. Y478C). Interestingly, for different types of mutations at the same amino acid site, effects on protein-protein interactions differ (R477 mutation) (Figure 3).

A previous study has shown that GC resistance can be caused by the intrinsic inhibition of apoptosis (Serafin et al., 2017; Brown and Ferrando, 2018). The selective pro-apoptotic response to GR signaling in lymphoid cells depends on their capacity to induce lymphoid-restricted transcriptional up-regulation of BCL2L11, which encodes the pro-apoptotic BH3-only factor BIM (Brown and Ferrando, 2018). This is consistent with our experimental results. Examining the expression of pro-apoptotic/anti-apoptotic genes, we revealed that NR3C1 mutations mainly up-regulated the anti-apoptotic genes (e.g., BCL2 and MCL1) in a dominant-negative manner, while the pro-apoptotic-related genes (e.g., *BAX, BAK, BID*, and *PUMA*) were non-dominantly negatively down-regulated. Therefore, mutations in the DBD of *NR3C1* can affect GC resistance via two mechanisms: dominant-negative regulation and non-dominant-negative regulation. Further investigation using RNA-seq and ChIP-seq technique is warranted to decipher the precise regulatory mechanisms and confirm our current findings.

## Conclusion and Perspective

In addition to somatic IKZF1, BTG1, TBL1XR1, and CREBBP lesions, NR3C1 also contributes to GC resistance and may contribute to relapse in children with ALL. Not all ALL-related *NR3C1* mutations have functional effects; however, the R477 amino acid residue is critical for normal function. NR3C1 mutations are more frequent in ALL cases with relapse than in newly diagnosed cases. In the future, deep sequencing to trace these acquired genetic mutations, permitting the early detection and treatment of relapse, may be warranted as another means to improve clinical outcomes.

## Data Availability

The datasets presented in this study can be found in online repositories. The name of the repository and accession number can be found below: Gene Sequence Archive for Human (GSA-Human) in National Genomics Data Center (NGDC), China National Center for Bioinformation (CNCB)/Beijing Institute of Genomics (BIG), https://bigd.big.ac.cn/gsa-human/, HRA000706.
