# Supplementary material for: Association Between NR3C1 Mutations and Glucocorticoid Resistance in Children With Acute Lymphoblastic Leukemia
Source: Front Pharmacol. 2021 Mar 29;12:634956. doi: 10.3389/fphar.2021.634956 (PMC8039513; doi:10.3389/fphar.2021.634956)
Supplement: Supplementary file 2 [file presentation1.pptx]

## Slide 1
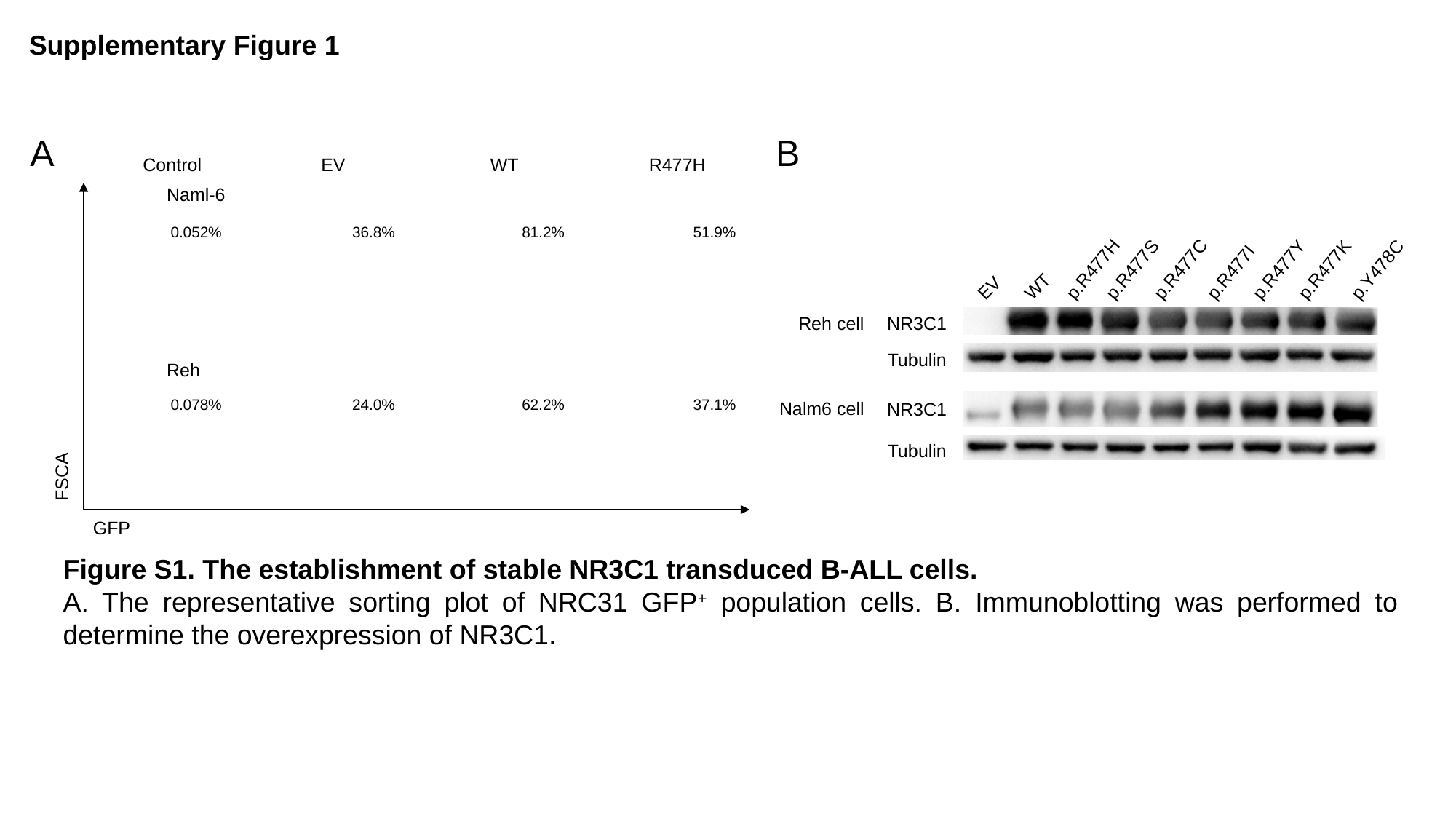

Supplementary Figure 1
A
B
Control
EV
WT
R477H
Naml-6
0.052%
36.8%
81.2%
51.9%
p.R477H
p.R477C
p.R477S
p.R477Y
p.R477K
p.Y478C
p.R477I
WT
EV
Reh cell
NR3C1
Tubulin
Reh
0.078%
24.0%
62.2%
37.1%
Nalm6 cell
NR3C1
Tubulin
FSCA
GFP
Figure S1. The establishment of stable NR3C1 transduced B-ALL cells.
A. The representative sorting plot of NRC31 GFP+ population cells. B. Immunoblotting was performed to determine the overexpression of NR3C1.

## Slide 2
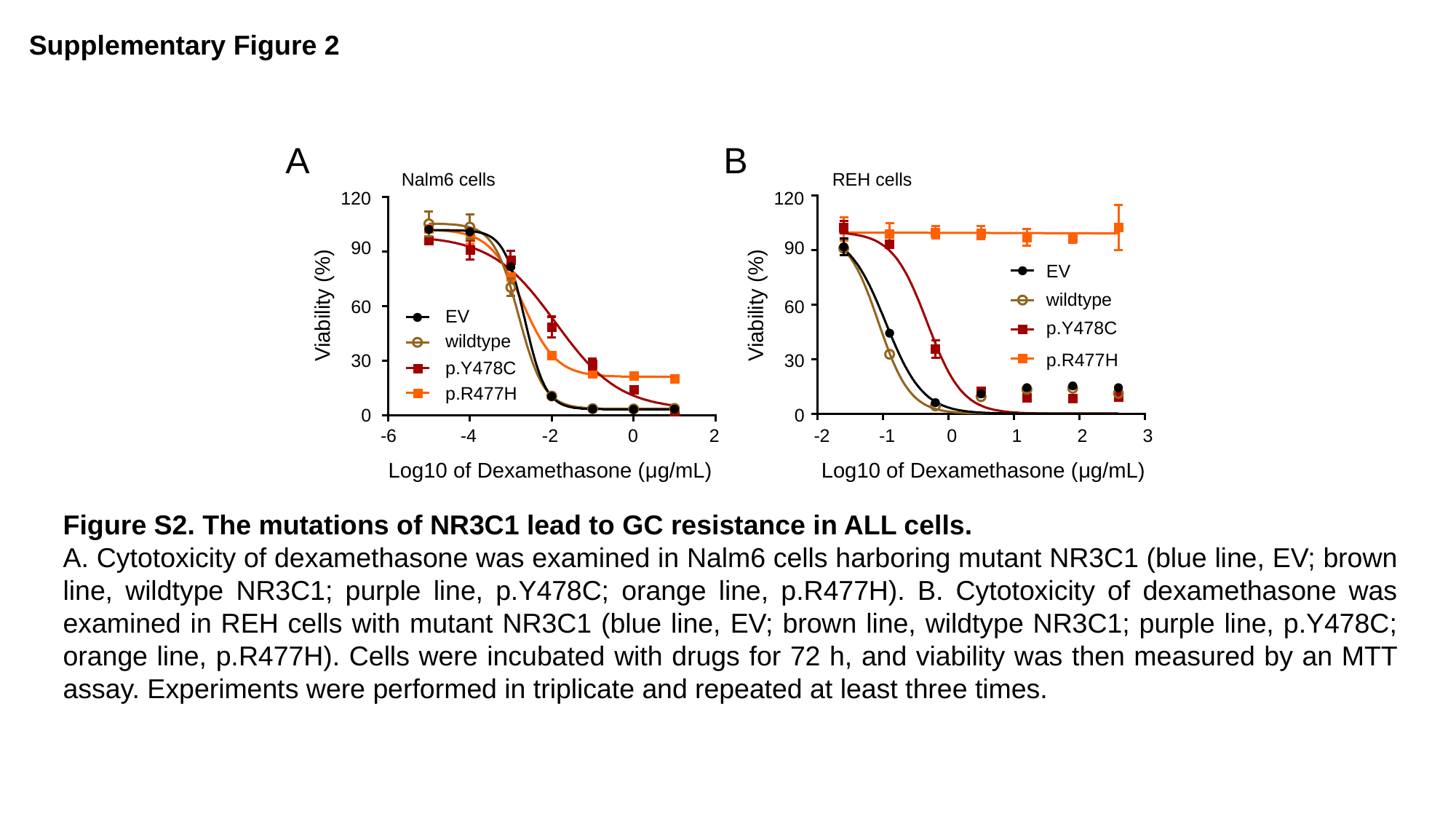

Supplementary Figure 2
A
B
Nalm6 cells
REH cells
120
90
EV
wildtype
Viability (%)
60
p.Y478C
p.R477H
30
0
-2
-1
0
1
2
3
Log10 of Dexamethasone (μg/mL)
120
90
Viability (%)
60
EV
wildtype
30
p.Y478C
p.R477H
0
-6
-4
-2
0
2
Log10 of Dexamethasone (μg/mL)
Figure S2. The mutations of NR3C1 lead to GC resistance in ALL cells.
A. Cytotoxicity of dexamethasone was examined in Nalm6 cells harboring mutant NR3C1 (blue line, EV; brown line, wildtype NR3C1; purple line, p.Y478C; orange line, p.R477H). B. Cytotoxicity of dexamethasone was examined in REH cells with mutant NR3C1 (blue line, EV; brown line, wildtype NR3C1; purple line, p.Y478C; orange line, p.R477H). Cells were incubated with drugs for 72 h, and viability was then measured by an MTT assay. Experiments were performed in triplicate and repeated at least three times.
